# Supplementary material for: Time-series transcriptome analysis identified differentially expressed genes in broiler chicken infected with mixed Eimeria species
Source: Front Genet. 2022 Aug 8;13:886781. doi: 10.3389/fgene.2022.886781 (PMC9393255; doi:10.3389/fgene.2022.886781)
Supplement: Supplementary file 2 [file DataSheet1.ZIP › 7dpi_GO.Gsea.1625106495923/GOBP_PROTEIN_O_LINKED_MANNOSYLATION.html]

Details for gene set GOBP\_PROTEIN\_O\_LINKED\_MANNOSYLATION[GSEA]

|  || Dataset | TMM\_7dpi\_gct\_format\_7dpi\_gct\_format.Class\_4dpi.cls #PC\_versus\_NC.Class\_4dpi.cls #PC\_versus\_NC\_repos |
| Phenotype | Class\_4dpi.cls#PC\_versus\_NC\_repos |
| Upregulated in class | 0 |
| GeneSet | GOBP\_PROTEIN\_O\_LINKED\_MANNOSYLATION |
| Enrichment Score (ES) | -0.7278962 |
| Normalized Enrichment Score (NES) | -1.8244793 |
| Nominal p-value | 5.3008215E-4 |
| FDR q-value | 0.10795908 |
| FWER p-Value | 0.8918 |
Table: GSEA Results Summary

  

Fig 1: Enrichment plot: GOBP\_PROTEIN\_O\_LINKED\_MANNOSYLATION      
 Profile of the Running ES Score & Positions of GeneSet Members on the Rank Ordered List

  

| SYMBOL | TITLE | RANK IN GENE LIST | RANK METRIC SCORE | RUNNING ES | CORE ENRICHMENT || 1 | DPM2 | na | 2774 | 0.154 | -0.2143 | No |
| 2 | POMGNT2 | na | 4221 | -0.020 | -0.3326 | No |
| 3 | TMTC1 | na | 5223 | -0.124 | -0.4024 | No |
| 4 | DPM1 | na | 6593 | -0.264 | -0.4875 | No |
| 5 | SDF2L1 | na | 6830 | -0.289 | -0.4754 | No |
| 6 | TMTC3 | na | 7344 | -0.346 | -0.4801 | No |
| 7 | POMT1 | na | 7794 | -0.394 | -0.4742 | No |
| 8 | FKTN | na | 10839 | -0.890 | -0.6301 | Yes |
| 9 | TMTC2 | na | 11100 | -0.964 | -0.5460 | Yes |
| 10 | POMT2 | na | 11103 | -0.965 | -0.4401 | Yes |
| 11 | LARGE1 | na | 11830 | -1.372 | -0.3500 | Yes |
| 12 | LARGE2 | na | 11894 | -1.492 | -0.1913 | Yes |
| 13 | TMTC4 | na | 11975 | -1.829 | 0.0029 | Yes |
Table: GSEA details [plain text format]

  

Fig 2: GOBP\_PROTEIN\_O\_LINKED\_MANNOSYLATION      
 Blue-Pink O' Gram in the Space of the Analyzed GeneSet

  

Fig 3: GOBP\_PROTEIN\_O\_LINKED\_MANNOSYLATION: Random ES distribution      
 Gene set null distribution of ES for **GOBP\_PROTEIN\_O\_LINKED\_MANNOSYLATION**
